# Association between voriconazole-induced visual hallucination and dopamine in an analysis of the food and drug administration (FDA) adverse event reporting system database

**DOI:** 10.1038/s41598-024-63504-y

**Published:** 2024-05-31

**Authors:** Hideo Kato, Chihiro Shiraishi, Mao Hagihara, Hiroshige Mikamo, Takuya Iwamoto

**Affiliations:** 1 Department of Pharmacy, Mie University Hospital, 174-2, Edobashi, Tsu, Mie, 514-8507 Japan; 2 Department of Clinical Pharmaceutics, Division of Clinical Medical Science, Mie University Graduate School of Medicine, Mie, Japan; 3 Department of Clinical Infectious Diseases, Aichi Medical University, 1-1, Yazakokarimata, Nagakute, Aichi 480-1195 Japan; 4 Department of Molecular Epidemiology and Biomedical Sciences, Aichi Medical University, 1-1, Yazakokarimata, Nagakute, Aichi 480-1195 Japan

**Keywords:** Voriconazole, Dopamine, Visual hallucination, Risk factors, Neurological manifestations, Infection

## Abstract

Voriconazole is a second-generation azole used to treat serious fungal infections. Visual hallucinations constitute a representative adverse event caused by voriconazole. However, its mechanism of action remains unclear. In patients with schizophrenia or Parkinson’s disease, the frequency of visual hallucinations is associated with brain dopamine levels. This study investigated the frequency of visual hallucinations in patients treated with voriconazole alone or in combination with dopaminergic medicines or dopamine antagonists, using data collected from the Food and Drug Administration Adverse event Reporting System (FAERS). The frequency of visual hallucinations with voriconazole alone and in combination with a dopaminergic medicine (levodopa) or dopamine antagonists (risperidone and chlorpromazine) was compared using data from the FAERS between 2004 and 2023, using the reporting odds ratio (ROR) with relevant 95% confidence intervals (CI). The reference group comprised patients who had been administered voriconazole without dopaminergic medication or dopamine antagonists. Of the patients, 22,839, 90,810, 109,757, 6,435, 20, 83, and 26, respectively were treated with voriconazole, levodopa, risperidone, chlorpromazine, voriconazole plus levodopa, voriconazole plus risperidone, and voriconazole plus chlorpromazine. The occurrence of visual hallucinations increased when used in combination with levodopa (ROR = 12.302, 95% CI = 3.587–42.183). No increase in incidence was associated with the concomitant use of dopamine antagonists (risperidone, ROR = 1.721, 95% CI = 0.421–7.030; chlorpromazine, ROR = none, 95% CI = none). Dopaminergic medicine may increase the risk of visual hallucinations in patients treated with voriconazole. Whether voriconazole positively modulates dopamine production warrants further investigation using a translational research approach.

## Introduction

Voriconazole is a broad-spectrum triazole antifungal agent, which is the first-line therapy for serious fungal infections, including invasive pulmonary aspergillosis^1^. Although voriconazole is a safe and well-tolerated drug, photophobia and visual hallucinations are common adverse events^2^. Once these adverse events occur, a dose reduction or discontinuation of voriconazole should be considered. A strong correlation has been observed between elevated trough concentrations of voriconazole and photophobia, whereas visual hallucinations have not been associated with voriconazole trough concentrations^3^. Therefore, the mechanisms by which visual hallucinations develop warrants further investigation.

Schizophrenia is a severe mental disorder characterized by visual hallucinations. The development and aggravation of schizophrenia are caused by increased dopamine levels in the brain^4^. Parkinson’s disease is a neurodegenerative disorder characterized by the loss of dopaminergic neurons in the brain^5^. The daily dose of levodopa and dopamine agonists in the treatment of Parkinson’s disease is remarkably higher in patients with visual hallucinations than in those without visual hallucinations^6^. Thus, visual hallucinations may be associated with dopamine hyperproduction. Additionally, we hypothesized that voriconazole treatment causes dopamine hyperproduction, which promotes the onset of visual hallucinations.

Spontaneous reporting systems are pivotal for investigating drug-drug interactions in patients prescribed multiple drugs. Whether a specific drug-drug combination moderates or enhances the frequency of adverse events has been identified using spontaneous reporting systems^7^. Therefore, we investigated the frequency of visual hallucinations in patients treated with voriconazole alone and in combination with dopaminergic medicines or dopamine antagonists, using data collected from the Food and Drug Administration (FDA) Adverse Event Reporting System (FAERS).

## Results

**Figure 1:**
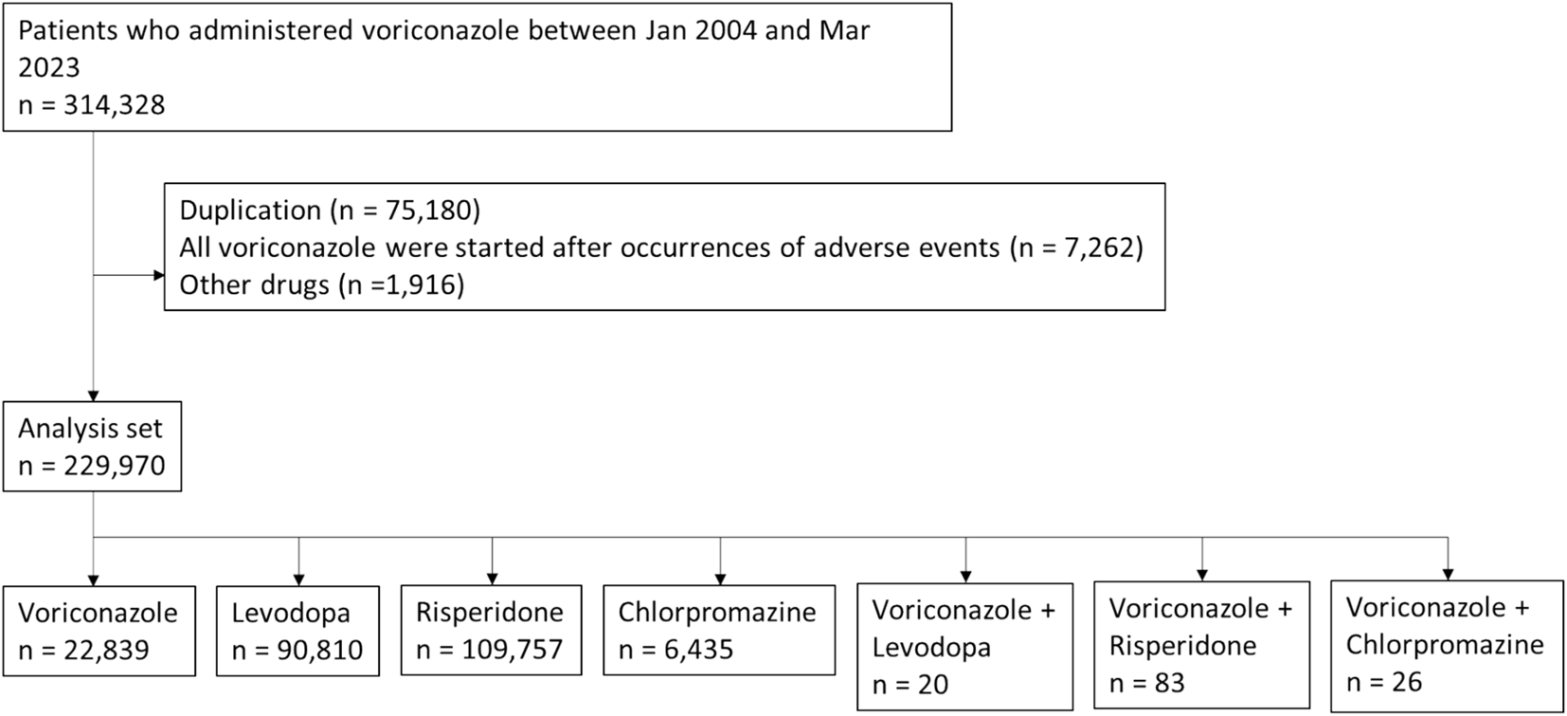
Flowchart of the data collection process.

Figure 1 shows a flowchart of the study. We retrieved 314,328 distinct patients who were administered voriconazole from January 2004 to March 2023. Of these, 75,180 duplicate cases, 7262 cases in which voriconazole was administered after the occurrence of adverse events, and 1916 cases in which other drugs were administered were excluded. The numbers of cases administered voriconazole, levodopa, risperidone, chlorpromazine, voriconazole plus levodopa, voriconazole plus risperidone, and voriconazole plus chlorpromazine were 22,839, 90,810, 109,757, 6,435, 20, 83, and 26.

The baseline characteristics of the patients from each group are shown in Table 1. Regarding sex distribution, the groups except for two groups (voriconazole + risperidone and voriconazole + chlorpromazine) consisted of more men than women. The median age of each group was 57, 73, 41, 47, 75, 66, and 41 years, respectively. Median weight of each group was 64.0, 70.3, 75.0, 70.0, 83.9, 49.0, and 53.0 kg. These cases mainly originated in the United States.

**Table 1:** Demographic characteristics of each patient in the FAERS database from January 2004 to March 2023.

	Voriconazole	Levodopa	Risperidone	Chlorpromazine	Voriconazole + Levodopa	Voriconazole + Risperidone	Voriconazole + Chlorpromazine
Sex, n (%)							
Female	7858 (34.4)	37,049 (40.8)	34,419 (31.4)	2712 (42.1)	8 (40.0)	41 (49.4)	18 (69.2)
Male	12,313 (53.9)	48,525 (53.4)	63,699 (58.0)	3143 (48.8)	11 (55.0)	35 (42.2)	7 (26.9)
Unknown	2668 (11.7)	5236 (5.8)	11,639 (10.6)	580 (9.0)	1 (5.0)	7 (8.4)	1 (3.8)
Age (years)							
Median [IQR]	57 [36–68]	73 [66–79]	41 [23–60]	47 [33–61]	75 [69–79]	66 [38–66]	41 [18–51]
Unknown, n (%)	4669 (20.4)	32,272 (35.5)	46,174 (42.1)	1302 (20.2)	1 (5.0)	12 (14.5)	1 (3.8)
Weight (kg)							
Medican [IQR]	64.0 [50.4–79.0]	70.3 [59.0–83.0]	75.0 [60.0–93.0]	70.0 [55.2–86.0]	83.9 [59.5–87.3]	49.0 [18.5–63.0]	53.0 [32.0–65.3]
Unknown, n (%)	16,581 (72.6)	68,060 (74.9)	86,925 (79.2)	4523 (70.3)	13 (65.0)	63 (75.9)	15 (57.7)
Country, n (%)							
United States	8729 (38.2)	45,740 (50.4)	62,347 (56.8)	1,978 (30.7)	8 (40.0)	39 (47.0)	9 (34.6)
France	2155 (9.4)	3389 (3.7)	6755 (6.2)	887 (13.8)	0 (0.0)	3 (3.6)	6 (23.1)
United Kingdom	1442 (6.3)	2804 (3.1)	5961 (5.4)	616 (9.6)	0 (0.0)	0 (0.0)	1 (3.8)
Germany	548 (2.4)	5773 (6.4)	4985 (4.5)	19 (0.3)	0 (0.0)	1 (1.2)	1 (3.8)
Japan	2426 (10.6)	3333 (3.7)	4623 (4.2)	726 (11.3)	5 (25.0)	12 (14.5)	2 (7.7)
Canada	543 (2.4)	1511 (1.7)	3691 (3.4)	261 (4.1)	0 (0.0)	2 (2.4)	0 (0.0)
Italy	518 (2.3)	2702 (3.0)	2546 (2.3)	447 (6.9)	0 (0.0)	1 (1.2)	0 (0.0)
Spain	633 (2.8)	2478 (2.7)	1450 (1.3)	48 (0.7)	0 (0.0)	19 (22.9)	0 (0.0)
Netherlands	444 (1.9)	2075 (2.3)	804 (0.7)	56 (0.9)	0 (0.0)	0 (0.0)	0 (0.0)
Australia	388 (1.7)	1339 (1.5)	1224 (1.1)	217 (3.4)	0 (0.0)	0 (0.0)	0 (0.0)
Others	4033 (17.7)	9503 (10.5)	10,112 (9.2)	728 (11.3)	7 (35.0)	3 (3.6)	4 (15.4)
Unknown	980 (4.3)	10,163 (11.2)	5259 (4.8)	452 (7.0)	0 (0.0)	3 (3.6)	3 (11.5)

Data are presented as medians [IQRs]. IQR, interquartile range.

The results of the disproportionality analyses of drug-drug interactions between voriconazole and dopaminergic medicine or dopamine antagonists are shown in Table 2. Visual hallucinations emerged with the use of levodopa alone (1.8%, reporting odds ratio (ROR) = 1.236, 95% CI = 1.096–1.394, *p* = 0.001). The occurrence of visual hallucinations induced by the concomitant use of voriconazole and levodopa was more frequently recorded than when voriconazole was used alone (17.6%, ROR = 12.302, 95% CI = 3.587–42.183, *p* < 0.001). Conversely, the use of risperidone or chlorpromazine alone was not associated with occurrence of visual hallucination (risperidone, 0.4%, ROR = 0.297, 95% CI = 0.258–0.343; chlorpromazine, 0.2%, ROR = 0.163, 95% CI = 0.097–0.274). The concomitant use of risperidone or chlorpromazine with voriconazole was not associated with an increased occurrence of voriconazole-induced visual hallucinations (voriconazole + risperidone, 2.5%, ROR = 1.721, 95% CI = 0.421–7.030; chlorpromazine, 0%; ROR, none; 95% CI, none).

**Table 2:** Disproportionality analyses and drug interaction approaches for various combinations involving voriconazole.

Exposure	Cases	Non-cases	ROR (95% CI)	*p*-value
Voriconazole	323	22,516	Reference	Reference
Levodopa	1582	89,228	1.236 (1.096–1.394)	0.001
Risperidone	466	109,291	0.297 (0.258–0.343)	< 0.001
Chlorpromazine	15	6420	0.163 (0.097–0.274)	< 0.001
Voriconazole + Levodopa	3	17	12.302 (3.587–42.183)	< 0.001
Voriconazole + Risperidone	2	81	1.721 (0.421–7.030)	0.449
Voriconazole + Chlorpromazine	0	26	–	–

## Discussion

This study used the worldwide open-access FAERS database to investigate the occurrence of visual hallucinations in patients who were administered voriconazole alone, concomitantly with dopaminergic medicines, or dopamine antagonists. Several findings emerged: (i) clinically relevant visual hallucinations occurred in patients who were administered voriconazole alone; (ii) as expected, dopaminergic medicine (levodopa) enhanced the incidence of visual hallucinations; and (iii) voriconazole did not increase the occurrence of visual hallucinations when combined with dopamine antagonists (risperidone and chlorpromazine).

Voriconazole can reach higher concentrations in the cerebrospinal fluid by traversing the blood–brain barrier^8^. Voriconazole binds to plasma proteins, and its concentration in the cerebrospinal fluid is about 30–60% of its concentration in plasma. A high concentration of voriconazole in the cerebrospinal fluid may cause changes in the retina and central nervous system, leading to photophobia and visual hallucinations^9^. However, a previous study suggested that a high concentration of voriconazole in the plasma may not increase the risk of visual hallucinations^3,10^. This issue requires further investigation.

Visual hallucinations are caused by continuous neurotransmission due to dopamine^11^. This is a low-sensitivity neurotransmission that responds only to transiently high concentrations of dopamine and does not respond to low concentrations of dopamine^11^. Our findings support a causal relationship between high levels of dopamine and the increased occurrence of visual hallucinations. Moreover, Yoshimura et al. reported changes in cerebral blood flow in two cases of visual hallucinations after meningioma excision^12^. They suggested that the pathogenesis of visual hallucinations involves an increase in cerebral blood flow, based on single-photon emission computed tomography analysis. However, no studies have reported on the association between voriconazole-induced visual hallucinations and altered cerebral blood flow, although cerebral blood flow increases in dogs receiving high dose of dopamine^13^. Therefore, increased cerebral blood flow caused by dopamine hyperproduction may be accompanied by visual hallucinations during voriconazole therapy.

Our study had inherent limitations associated with the analysis of spontaneous reporting data: lack of exposure data, inability to firmly infer causality, and explanation for all potential confounders^14^. Therefore, although the cases of levodopa use included a large number of older adults, it was difficult to discuss the impact of different patient backgrounds. However, the FAERS is representative of the worldwide real-life use of medicines, which cannot be accurately represented by clinical trials^15^. In fact, no patients were administered both voriconazole and levodopa, risperidone, or chlorpromazine at our hospital. Moreover, despite these limitations, FAERS has a large sample size and is suitable for discovering the mechanisms of rare drug-drug interactions. Next, the group with the concomitant use of voriconazole and levodopa was small sample size. Hence, the precision of statistics would be lack. However, it has been reported that results of the Bayesian measures are similar when the reported count is more than three^16^. Finally, the association with dopamine was inferred based on dopaminergic medicines and dopamine antagonists and not based on actual dopamine levels. In the future, basic or clinical studies should further investigate the signals detected by FAERS.

## Conclusion

Dopaminergic medicine may increase the risk of visual hallucinations in patients treated with voriconazole. Clinicians should remain vigilant concerning the occurrence of visual hallucinations in patients administered with voriconazole, especially in those treated with dopaminergic medicine. However, there is still the possibility that concomitant use with levodopa is simply an additive increase in the risk unrelated to dopamine. Therefore, further investigation with a translational research approach to reveal whether voriconazole positively modulates dopamine production or not warrants our study results.

## Materials and methods

### FAERS database

The FAERS database contains over 1.4 million cases of spontaneous adverse events submitted by pharmaceutical companies, clinicians, pharmacists, and patients. It contains the following types of data: patient demographics, administrative information, drug names, adverse events, patient outcomes, report sources, therapy dates, and indications for use^17^. Informed consent was not required since our study was based on a public database. Adverse event reports between January 2004 and March 2023 were extracted from the FAERS database. Reports with the same number of cases were identified as duplicate reports, and the most recent reports were used in accordance with to FDA recommendations^18^. Since occurrence of visual hallucination was not caused by voriconazole in cases in which voriconazole was administered after the occurrence of adverse events, the reports were excluded. We collected reports on the most widely used dopaminergic medicine (levodopa which mainly acts as dopamine transformed within brain) and antagonists (risperidone and chlorpromazine). The classification and standardization of adverse events in the FAERS database are based on the Medical Dictionary for Regulatory Activities (MedDRA)^19^. In the FAERS database, each report is coded using preferred terms from the MedDRA terminology. The collected data included case numbers, drug names, adverse event names, sex, and age derived from the MedDRA terminology.

### Definition of visual hallucination

Visual hallucinations were coded according to the preferred terms (10047570) derived from MedDRA terminology. Event reports were identified using a standardized MedDRA query (version 26.0). In this study, visual hallucinations were defined as adverse events. All other cases without visual hallucinations were considered non-cases.

### Data analysis

In spontaneous reporting databases, possible interactions are detected based on the demonstration that a suspected adverse event has been reported more frequently with a combination of two drugs than when they are used alone^20^. The reports were divided into three index groups: (i) reports of patients exposed to voriconazole alone, (ii) reports of patients exposed to dopaminergic medicine or antagonists, and (iii) reports of patients exposed to both voriconazole and dopaminergic medicine or antagonists at the time of the event. The reference group consisted of patients administered voriconazole. Disproportionality was calculated using the ROR with relevant 95% confidence intervals (CIs), which were defined as statistically significant when the lower limit of the 95% CI exceeded 1, with at least three cases of interest reported^16^. Statistical analyses were performed using R software version 4.1.3 (R Core Team, 2022).

## Data Availability

Data are available from the corresponding author upon reasonable request. The FAERS datasets analyzed during the current study are available on FDA websites (https://www.fda.gov/).

## Abbreviations

FAERS: Food and drug administration adverse event reporting system
CI: Confidence interval
ROR: Reporting odds ratio
